# Nectin-1 and HVEM: cellular receptors for HSV-1 in skin

**DOI:** 10.18632/oncotarget.8340

**Published:** 2016-03-24

**Authors:** Dagmar Knebel-Mörsdorf

**Affiliations:** Center for Biochemistry, University of Cologne, Cologne, Germany

**Keywords:** viral receptors, herpes simplex virus, skin

Herpes simplex virus (HSV) can cause a range of diseases in humans from mild uncomplicated mucocutaneous lesions to life-threatening infections. HSV 1 (HSV-1) is dominantly associated with orofacial infections and encephalitis, and can be devastating in immune-compromised hosts and newborns. Although entry into individual cells in culture and the immune response to HSV-1 have been studied in detail, we have little knowledge of how the virus invades actual target tissues or of which cellular factors determine susceptibility to infection *in vivo.* Two important factors are the cell surface receptors, herpesvirus entry mediator (HVEM) and nectin-1, which interact with the viral envelope glycoprotein D (gD) [1]. This interaction is essential for the fusion of the viral envelope with cellular membranes, allowing delivery of the nucleocapsid into the cytoplasm to initiate infection. Target tissues for HSV-1 in the human host are mucosal surfaces, skin and cornea. Mice are widely used as an animal model to investigate HSV pathogenesis, since the murine homologs of nectin-1 and HVEM also act as efficient receptors and the presence of either nectin-1 or HVEM is sufficient for disease development in mice [2]. Although gD interactions with HVEM or nectin-1 have been studied extensively in different cell lines, the contributions of the individual receptors to uptake into tissue are not well understood.

We established an *ex vivo* infection model of murine epidermal sheets to analyze the contribution of the receptors, and used an experimental setting that enables the virus to enter *via* the basal layer of the epidermis. Infection studies in HVEM- or nectin-1-deficient epidermis identified nectin-1 as the major receptor in the epidermal sheets, while HVEM had a more limited role [3]. Keratinocytes are the major cell type in the epidermis and when cultured murine primary keratinocytes that expressed neither HVEM nor nectin-1 were examined, almost no infected cells were observed [3]. Since the epidermis represents only the outermost layer of skin, we also addressed the contribution of nectin-1 and HVEM as receptors in the underlying dermis. Fibroblasts are the major resident cell type of the dermis. When we infected murine primary dermal fibroblasts which were deficient in nectin-1, infection was slower, suggesting that HVEM is a less efficient receptor. In the absence of both HVEM and nectin-1, infection was severely delayed resulting in greatly reduced viral spreading and virus production [4]. In contrast to cultured keratinocytes, there was residual infection suggesting the presence of a further, rather inefficient receptor.

Comparison of the two major cell types of skin, keratinocytes in the epidermis and fibroblasts in the underlying dermis, demonstrated that nectin-1 is less highly expressed on fibroblasts than on keratinocytes. In contrast, HVEM is present on nearly all fibroblasts but only expressed on a few keratinocytes in epidermis. Interestingly, these expression levels do not appear to correlate with their effectiveness as receptors. Despite its low level on fibroblasts, our results support nectin-1 as the major mediator of HSV-1 entry into both cell types ofmurine skin [3, 4]. In the absence ofnectin-1, HVEM can replace it as a receptor, and appears to do so more efficiently in fibroblasts than in keratinocytes.

Nectin-1 is a Ca^2+^-independent cell-cell adhesion molecule involved in the formation of adherens junctions, and is expressed throughout the murine epidermis [5]. HVEM is a member of the tum or necrosis factor receptor family and can activate either pro-inflammatory or inhibitory signaling pathways [6]. Thus, the differential contribution of nectin-1 and HVEM to efficient entry of HSV-1 into skin might reflect differing outcomes of receptor binding. It seems clear that nectin-1 binding accounts primarily for the uptake mechanism, while HVEM binding may have a secondary effect of modulating the immune response by interfering with natural ligands. The rapid loss of nectin-1 from the surface of epidermal keratinocytes and dermal fibroblasts upon infection supports this assumption [3, 4]. A further intriguing question is whether and how HSV-1 gains access to the cell-cell adhesion molecule nectin-1 in intact skin or mucosa where close cell-cell contacts might be expected to act as a barrier.

Characterization of the uptake pathway in murine skin suggests that HSV-1 enters into epidermal sheets, primary epidermal keratinocytes and primary dermal fibroblasts, both by direct fusion of the viral envelope with the plasma membrane and *via* endocytic vesicles [3, 4]. Interestingly, this is not dependent on the presence or absence of nectin-1, suggesting that nectin-1 and HVEM can initiate both uptake modes. Whether both internalization pathways lead to productive infection is difficult to determine although studies in human keratinocytes support endocytic uptake as contributing to HSV-1 entry [7]. In addition, we demonstrated that entry into skin cells is cholesterol- and dynamin-m ediated [4, 7]. Based on the known functions of dynamin, the finding that inhibition of dynamin GTPase activity results in a complete block of uptake, was unexpected [7]. A likely explanation is that both the fusion events at the plasma membrane and vesicle scission depend on dynamin.

In these studies, we have demonstrated the involvement of cellular receptors during HSV-1 entry into murine epidermis and compared the entry pathways into the two major cell types of skin. This approach will allow us to transfer our know ledge of virus entry mechanisms resulting from studies in various cell lines into an understanding of how HSV enters its natural target tissues. In addition, it provides a means to explore how HSV overcomes the barrier functions of skin and mucosa to reach its receptors and initiate infection.
